# Implications of autolysosome- astrocyte-associated signature in the pathogenesis of Alzheimer’s disease: evidence from artificial intelligence and multi-omics and clinical validation

**DOI:** 10.3389/fnins.2026.1867831

**Published:** 2026-07-02

**Authors:** Congmin Zhang, Dandan Song

**Affiliations:** Department of Pharmacy, Shaoxing Seventh People’s Hospital (Affiliated Mental Health Center, Shaoxing University), Shaoxing, Zhejiang, China

**Keywords:** Alzheimer’s disease, artificial intelligence, astrocytes, autolysosome, multi-omics

## Abstract

**Background:**

Alzheimer’s disease (AD) is a progressive neurodegenerative disorder characterized by amyloid-beta plaques and neurofibrillary tangles. Dysfunctional cellular clearance mechanisms, particularly autophagy-lysosomal pathways, and reactive astrocytosis are prominent pathological features, yet their interrelationship remains poorly defined.

**Objective:**

This study aimed to decipher a novel co-expression molecular signature linking autolysosomal dysfunction and astrocyte reactivity in AD pathogenesis.

**Methods:**

We performed Limma, WGCNA and Xcell algorithms in AD patient hippocampus bulk profiles for enrichment of astrocyte and autolysosome (AA)-associated DEGs. Next, explainable machine learning and consensus clustering enables the identification of AA-associated diagnostic model and molecular subgroups for AD patients at bulk level. Besides, AA-associated central pathogenic factor was identified, and its corresponding biological implications for AD were assessed at AD patient hippocampus single-cell level in temporal and spatial manners. Next deep learning algorithm (Drugreflector) and molecular docking enriched natural compounds for the treatment of AD by targeting AA-associated hub gene. Finally, AD clinical peripheral blood samples were collected for estimation of hub gene expression patterns.

**Results:**

5 AA-associated shared DEGs can elaborate diagnostic and patient stratification capacity for AD patients. HMGCR can be considered as astrocyte-distributed central pathogenic and Berberine-oriented therapeutic target for AD patients.

**Conclusion:**

Our findings unveil AA-associated diagnostic model and molecular subgroups coupled with HMGCR center pathogenic and druggable role in AD, which represents an actionable clinical target for AD patients.

## 1 Introduction

Alzheimer’s disease (AD) stands as the most prevalent cause of dementia, imposing a tremendous global health burden (Lane et al., 2018). Pathologically, it is defined by the extracellular accumulation of amyloid-β (Aβ) plaques and intracellular neurofibrillary tangles composed of hyperphosphorylated tau protein (Twarowski and Herbet, 2023). These hallmarks trigger a cascade of events including synaptic loss, chronic neuroinflammation, and eventual neuronal death, leading to progressive cognitive decline (Lopez-Lee et al., 2024). Despite decades of research, the complex interplay between various cellular pathways contributing to AD progression is not fully understood, highlighting the need to identify novel pathogenic axes and therapeutic targets (Hardy, 2025).

Among the myriad of dysregulated processes in AD, two have garnered significant attention: impaired autophagy-lysosomal function and astrocyte reactivity (Carter et al., 2019; Mançano et al., 2024). The autophagy-lysosomal pathway is the primary mechanism for degrading protein aggregates and damaged organelles (Carter et al., 2019). Its dysfunction, leading to the accumulation of aberrant autolysosomes, is increasingly recognized as an early and critical event in AD, contributing to Aβ and tau pathology (Nixon and Rubinsztein, 2024). Concurrently, astrocytes, the most abundant glial cells in the brain, undergo profound morphological and functional changes in AD, a state known as reactive astrocytosis (Wu and Eisel, 2023). While initially protective, chronic astrocyte reactivity can become detrimental, exacerbating neuroinflammation and synaptic dysfunction (Ziar et al., 2025). Intriguingly, emerging evidence suggests a bidirectional relationship between lysosomal health and astrocyte function, but the specific molecular mediators of this autolysosome-astrocyte (AA)-related signature remain largely unexplored.

In this study, we aimed to define AA-associated molecular signature exists and drives key aspects of AD pathogenesis. To investigate this, we conducted a comprehensive, artificial intelligence (AI)-augmented multi-omics analysis integrating bulk tissue transcriptomics, single-cell resolution data, and clinical validation. Our objectives were to identify and characterize a core AA-associated gene signature for assessment of its associated diagnostic and molecular subtypes coupled with exploration of potential as a therapeutic target. This work aims to provide a novel framework for understanding AD pathology through the lens of glial-lysosomal crosstalk.

## 2 Material and methods

### 2.1 Source of bulk data

Bulk RNA-sequencing datasets derived from human hippocampal tissue were retrieved from the GEO database. GSE36980 (internal set 1) included 8 AD and 10 control samples (Zhu et al., 2022). GSE28146 (internal set 2) comprised 8 control and 22 AD samples (Wang et al., 2022). GSE29378 (training set) contained 32 control and 31 AD samples, while GSE48350 (validation set) included 43 control and 19 AD samples (Miao et al., 2025; Wang et al., 2024). Raw data were normalized using the limma package in R (Ritchie et al., 2015). Batch effects were corrected using the ComBat algorithm from the sva package in R (Leek et al., 2012). A curated list of genes of autolysosome-related genes was compiled from the Genecards database with threshold greater than 1.

### 2.2 Limma analysis

Differential expression analysis between AD and control samples in GSE36980 was performed using the limma package in R (Ritchie et al., 2015). Genes meeting the threshold of | log2 fold change (FC)| > 0.5 and a *p* < 0.05 were designated as DEGs (Ritchie et al., 2015). This DEG list was intersected with the autolysosome-related gene set to identify autolysosome-associated DEGs. The expression pattern of these genes was visualized with a heatmap using the pheatmap package in R (Zhang X. et al., 2023). Functional enrichment analysis GO and KEGG analysis was conducted using the clusterProfiler package (v4.2.2) in R in reference to the GO and KEGG gene set downloaded from MSIGDB database (Yu et al., 2012).

### 2.3 WGCNA analysis

The relative abundance of astrocytes in the hippocampal microenvironment of GSE28146 was estimated using the xCell algorithm in R (Aran et al., 2017). To identify co-expression modules correlated with astrocyte abundance, a WGCNA was performed using the WGCNA package in R (Langfelder and Horvath, 2008). An appropriate soft-thresholding power (β) was selected to achieve a scale-free topology fit index (R^2^) (Langfelder and Horvath, 2008). The TOM was constructed, and modules were identified via hierarchical clustering with a minimum module size of 30 genes(Langfelder and Horvath, 2008). The module eigengene exhibiting the strongest correlation (Pearson) with the estimated astrocyte score was defined as the astrocyte-associated module (Langfelder and Horvath, 2008). Genes from this module were extracted and intersected with the autolysosome-associated DEGs to define the AA-associated shared DEGs.

### 2.4 Explainable machine learning model construction

A machine learning framework was implemented using the caret package in R to build a diagnostic model based on the AA-associated shared DEGs (Wu et al., 2025). A total of 132 model combinations were trained and optimized on the GSE29378 training set using 10-fold cross-validation repeated 5 times (Wu et al., 2025). The model with the highest mean area under the receiver operating characteristic curve (AUC-ROC) was selected (Wu et al., 2025). Its performance was rigorously evaluated on the independent validation set GSE48350 (Wu et al., 2025). Model calibration was assessed via calibration plots, and a nomogram was constructed using the rms package in R in GSE29378 (Wu et al., 2025). To interpret the model and identify the most influential feature, SHAP analysis in R was performed (Zhou et al., 2026).

### 2.5 Consensus clustering analysis

Based on the expression profiles of the AA-associated shared DEGs, consensus clustering was applied to the AD samples in GSE29378 using the ConsensusClusterPlus package in R (Hu et al., 2022). The optimal number of clusters (k) was determined by evaluating the consensus matrix heatmap and the cumulative distribution function (CDF) curve (Hu et al., 2022). Samples were assigned to molecular subtypes accordingly (Hu et al., 2022). Differences in the expression of the AA signature genes and immune cell infiltration via CIBERSORT package in R between subtypes were compared. GSEA was performed to identify differentially activated pathways between the subtypes via clusterProfiler package in R in reference to the GO and KEGG gene set downloaded from MSIGDB database (Huang et al., 2024; Yu et al., 2012).

### 2.6 Single-cell analysis

GEO-acquired single-cell RNA sequencing data from AD hippocampal tissues (GSE163577, *n* = 9 AD samples) were processed using the Seurat package in R (Butler et al., 2018; He et al., 2022). Cells with unique feature counts <200 or > 6,000, or with > 15% mitochondrial gene content was filtered out (Butler et al., 2018). Data were normalized, and the top 2,000 highly variable genes were identified (Butler et al., 2018). PCA was followed by graph-based clustering and visualization with UMAP and t-SNE (Butler et al., 2018). Cell types were annotated using the SingleR package in R with the Human Primary Cell Atlas as a reference (Aran et al., 2019). Cell-cell communication analysis was performed using CellphoneDB package in R (Efremova et al., 2020). Metabolic heterogeneity was assessed via the scMetabolism package in R (Argüello et al., 2020). Autolysosome pathway activity was scored across cell types using the AUCell package in R in accordance with the autolysosome-associated gene list (Deng et al., 2021). A virtual knockout of the hub gene in the astrocyte cluster was simulated using the scTenifoldKnk package in R, and the perturbed genes were subjected to functional enrichment analysis via KEGG and GO analysis in reference to the GO and KEGG gene set downloaded from MSIGDB database (Osorio et al., 2022). Pseudotime trajectory analysis of astrocytes and expression trajectory of targeted gene in astrocyte was conducted using Monocle2 package in R (Fang et al., 2022).

### 2.7 Drug screening

The AI-based platform DrugReflector was employed for drug repurposing screening. The bulk expression signature of GSE29378 was used as a query against the TCMSP database (DeMeo et al., 2025). Compounds with predicted inverse correlation profiles were ranked by calculating drug-target association score(DTS) for achieving greater than 0.9 (DeMeo et al., 2025). The top candidate underwent molecular docking studies (DeMeo et al., 2025). The 3D protein structure of targeted genes was retrieved from the RCSB PDB, and the structure of targeted compound was obtained from PubChem database (Xu et al., 2023). Docking simulations were performed using AutoDock Vina software, and the binding pose was visualized with PyMOL software (Xu et al., 2023).

### 2.8 Clinical sample validation

Peripheral blood samples from 10 AD patients and 10 age-matched healthy control controls were obtained from Department of Pharmacy, Shaoxing Seventh People’s Hospital. The use of human tissue was approved by the local ethics committee, and informed consent was obtained from all donors. Total RNA was extracted using TRIzol™ Reagent (Invitrogen, Thermo Fisher Scientific, United States) strictly according to the manufacturer’s instructions, and its concentration and purity were assessed using a NanoDrop™ One spectrophotometer (Thermo Fisher Scientific, United States). Subsequently, 1 μg of total RNA was reverse-transcribed into cDNA using the PrimeScript™ RT reagent Kit with gDNA Eraser (Takara Bio, Japan) to eliminate genomic DNA and synthesize first-strand cDNA. Quantitative PCR was performed on a QuantStudio™ 5 Real-Time PCR System (Applied Biosystems, United States) using reaction mixtures prepared with TB Green^®^ Premix Ex Taq™ II (Tli RNaseH Plus) (Takara Bio, Japan). The sequences of the specific primers used for amplifying HMGCR and the reference gene GAPDH were as follows.

HMGCR:

F: 5’-AGCTTGCCCAGATTCTCATCC-3’

R: 5’-TCTGCCATAAGCACGTAGTCC-3’

GAPDH:

F: 5’-GGAGCGAGATCCCTCCAAAAT-3’

R: 5’-GGCTGTTGTCATACTTCTCATGG-3’

### 2.9 Statistical analysis

All statistical analyses were performed using R (v4.2.2) or GraphPad Prism (v9.0). For comparisons between two groups, Student’s *t*-test (for normally distributed data) or the Mann-Whitney U test (for non-normally distributed data) was applied. For comparisons among multiple groups, one-way ANOVA followed by Tukey’s post-hoc test was used. Correlation analyses were performed using Pearson or Spearman methods. A *p* < 0.05 or FDR < 0.05 was considered statistically significant.

## 3 Results

### 3.1 Identification of autolysosome-related DEGs for AD patients

**FIGURE 1:**
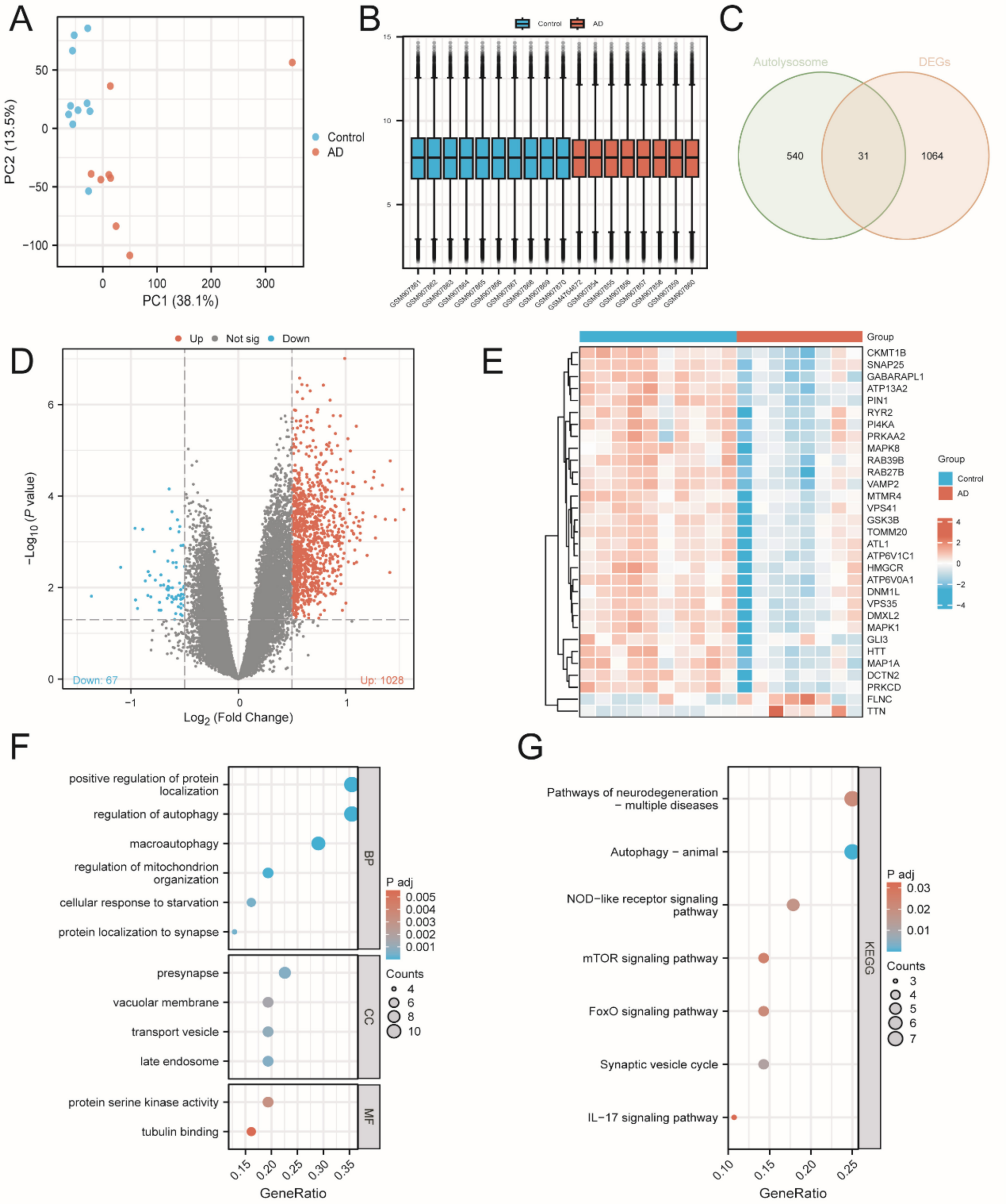
Identification of autolysosome-associated DEGs in AD hippocampus. **(A)** PCA plot of GSE36980. **(B)** Box plot of GSE36980. **(C)** Venn diagram showing the intersection of DEGs with autolysosome-related genes. **(D)** Volcano map of DEGs in GSE36980. **(E)** Heatmap of the 31 autolysosome-associated DEGs. **(F,G)** GO and KEGG pathway enrichment analysis of the 31 DEGs.

Analysis of the GSE36980 hippocampal dataset identified 1,095 DEGs between AD and control samples. Intersection with a curated autolysosome gene set yielded 31 autolysosome-associated DEGs (Figures 1A–D). A heatmap demonstrated a distinct expression pattern of these 31 genes in AD versus control hippocampus (Figure 1E). GO and KEGG enrichment analysis revealed these genes were significantly involved in Autophagy, Lysosome, and mTOR signaling pathway (Figures 1F,G), confirming their relevance to the autophagy-lysosomal system.

### 3.2 Identification of astrocyte-related DEGs for AD patients

**FIGURE 2:**
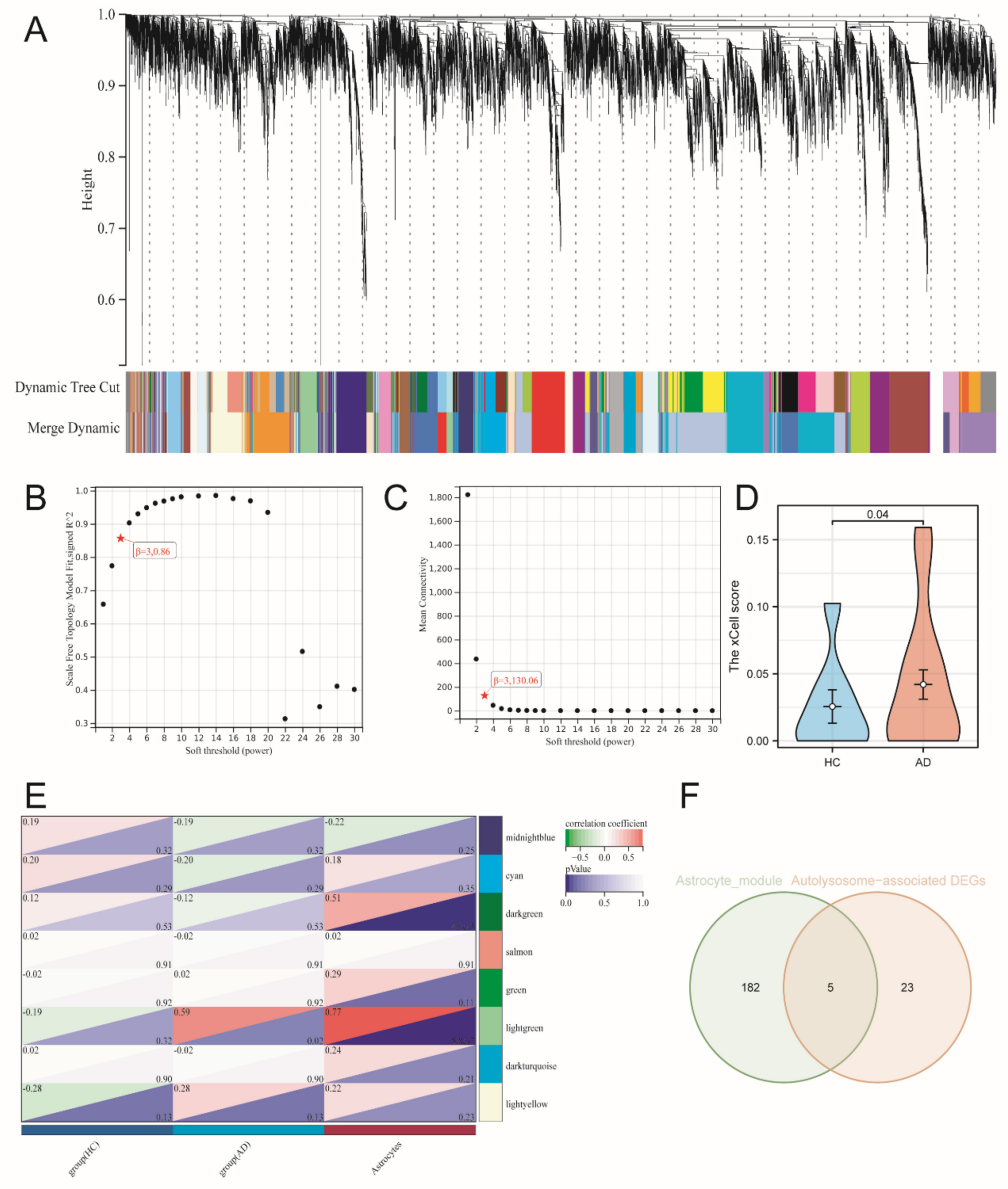
WGCNA identifies an astrocyte-associated module and defines the AA-associated gene signature. **(A)** Denogram cutting tree of WGCNA analysis. **(B,C)** β threshold of WGCNA analysis. **(D)** xCELL analysis of astrocyte infiltration. **(E)** Module-trait correlation heatmap. **(F)** Venn diagram illustrating the intersection leading to the 5-gene AA-associated signature.

xCELL analysis of GSE28146 revealed a significant increase in estimated astrocyte abundance in AD hippocampal samples compared to controls (*p* < 0.01) (Figure 2D). WGCNA identified 8 co-expression modules with β threshold as 3,086 and 3,130.06 (Figures 2A–C,E). The lightgreen module showed the strongest positive correlation with both disease status and astrocyte abundance (Figure 2E). This module contained 187 genes. The intersection of genes from the lightgreen module with the 31 autolysosome-associated DEGs yielded a core 5-A) signature (Figure 2F).

### 3.3 Identification of AA-associated diagnostic model for AD patients

**FIGURE 3:**
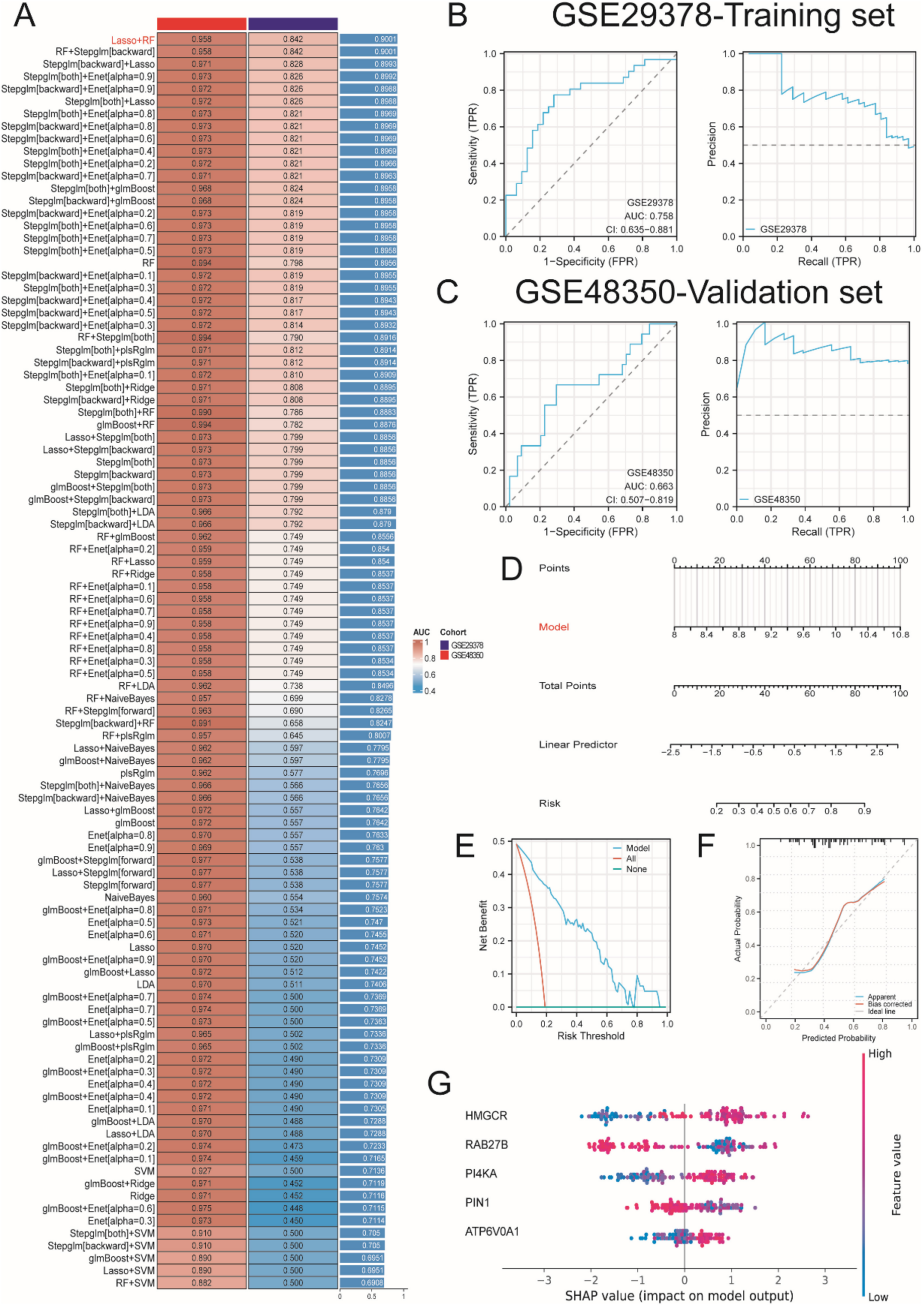
Development and validation of the AA-associated diagnostic model for AD patients. **(A)** Systemic machine learning model with 132 combinations in GSE29378 and GSE48350. **(B)** ROC and PR curves of GSE29378 with AUC of ROC = 0.758. **(C)** ROC and PR curves of GSE48350 with AUC of ROC = 0.663. **(D–F)** Nomogram analysis of model examination in GSE29378. **(G)** SHAP analysis of AA-associated shared DEGs in machine learning model construction.

A machine learning framework evaluated 132 model combinations on the training set (GSE29378). The Random Forest with Lasso model demonstrated superior and stable performance, achieving an AUC of 0.758 in training set (Figures 3A,B). This high diagnostic accuracy was successfully validated in the independent cohort GSE48350(AUC = 0.663) (Figure 3C). The nomogram and calibration curve indicated excellent agreement between predicted and observed probabilities of AD in the training set (Figures 3D–F). SHAP analysis, which quantifies the contribution of each feature to the model output, unequivocally identified HMGCR as the most influential gene for distinguishing AD from controls (Figure 3G).

### 3.4 Identification of AA-associated molecular subgroups for AD patients

**FIGURE 4:**
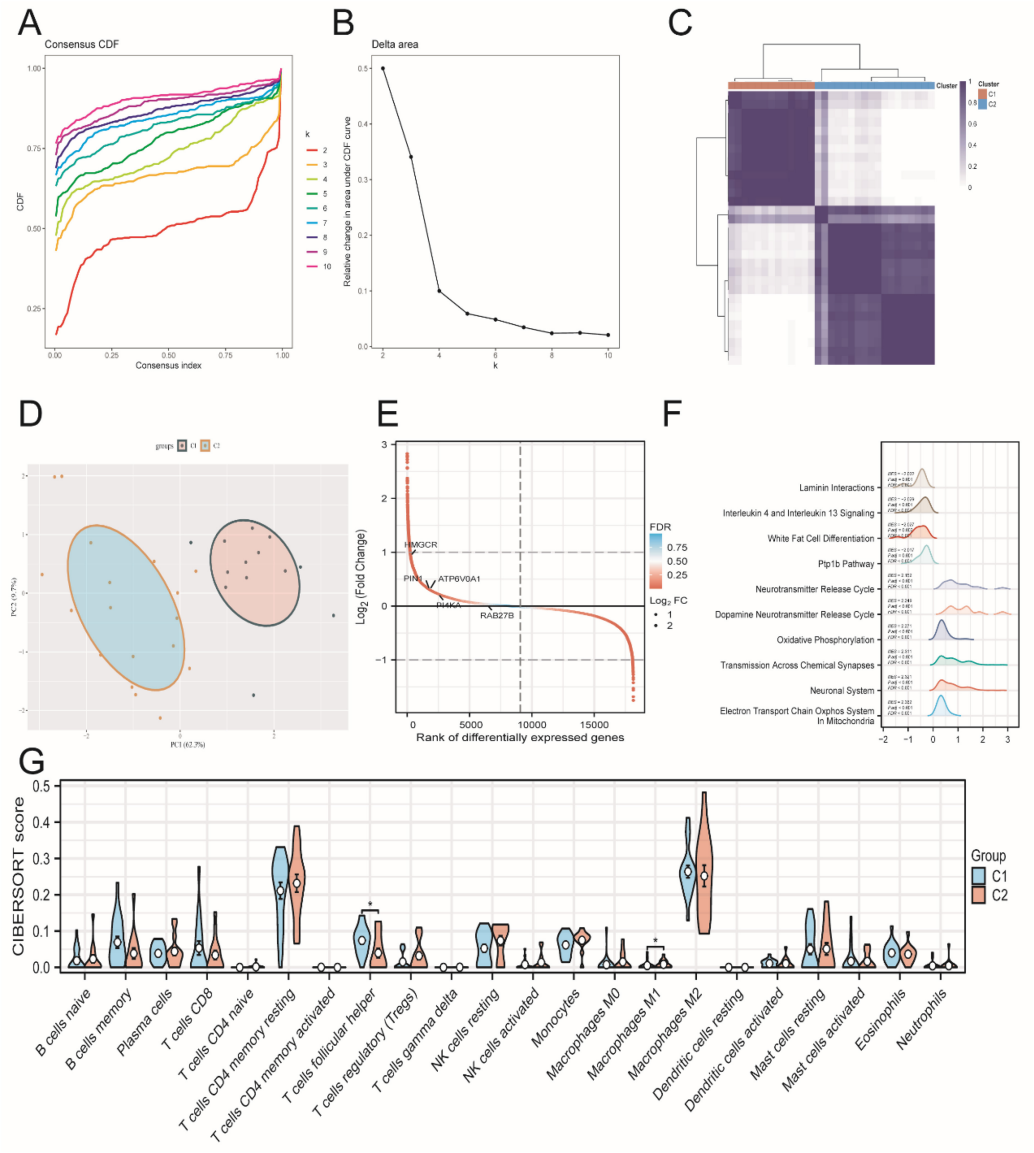
Molecular subtyping of AD patients based on the AA-associated signature. **(A–C)** Consensus matrix heatmap for *k* = 2. **(D)** PCA plot showing the separation of C1 and C2 subtypes. **(E)** Heatmap comparing the expression of the 5 AA signature genes between subtypes. **(F)** GSEA plots showing representative enriched pathways in C1 vs. C2. **(G)** Box plot of immune cell infiltration differences between C1 and C2 subtypes.

Consensus clustering based on the 5-gene AA signature robustly stratified AD patients in GSE29378 into two distinct molecular subtypes, designated C1 and C2 (Figures 4A–D). The expression of all five AA signature genes, particularly HMGCR, was significantly higher in the C1 subtype (Figure 4E). GSEA revealed that the C1 subtype was enriched in activation of neurotransmitter release cycle and inhibition of functions related to autophagy (Figure 4F). Correspondingly, CIBERSORTx analysis indicated a markedly different immune microenvironment, with the C1 subtype exhibiting higher scores for T follicular helper and macrophage infiltration (Figure 4G).

### 3.5 Single-cell atlas of astrocyte in AD patients

**FIGURE 5:**
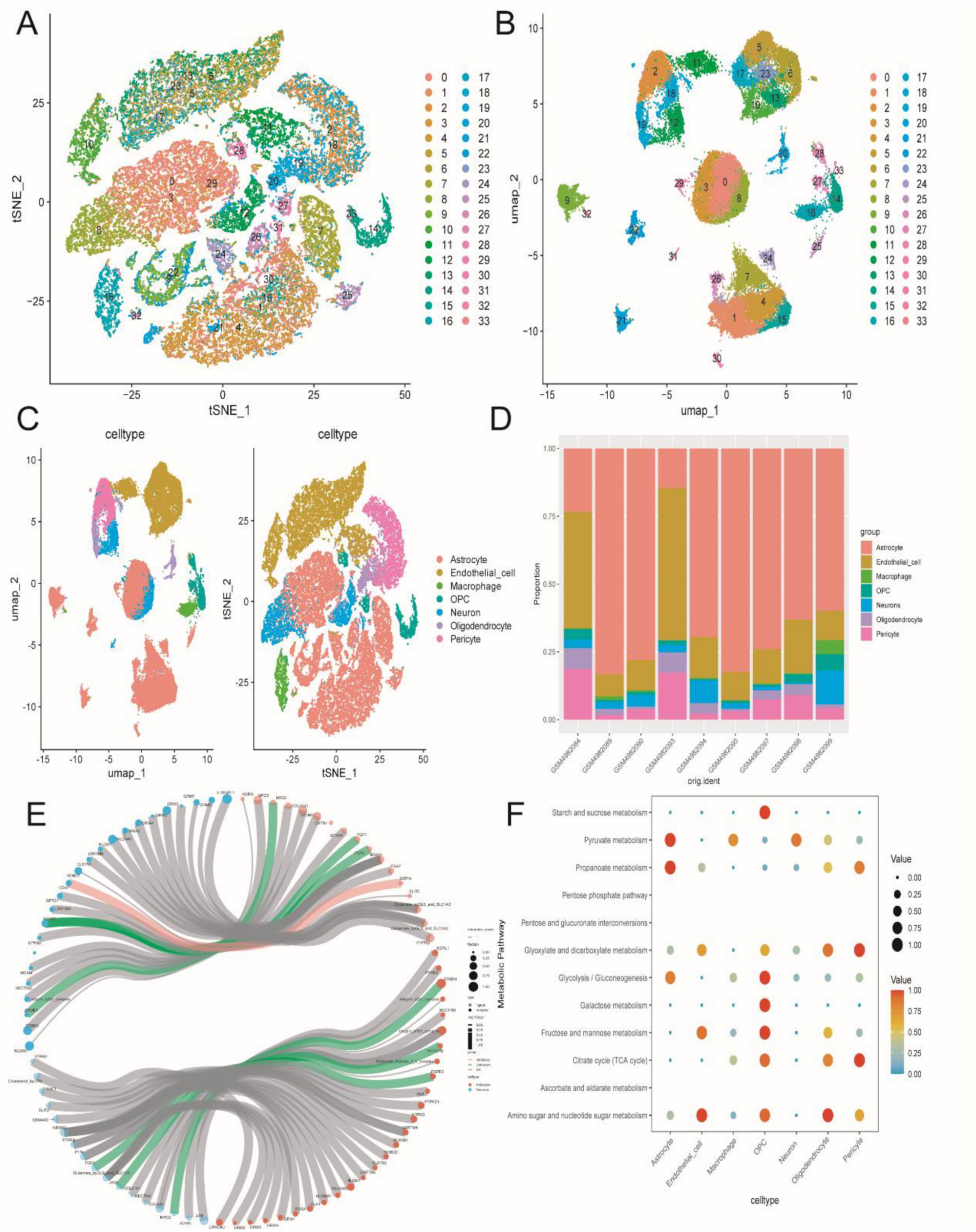
Single-cell landscape of the AD hippocampus and corresponding astrocyte interaction and metabolic patterns. **(A,B)** UMAP and t-SNE plots of all cells colored by cluster. **(C)** T-SNE and UMAP plot annotated by major cell types. **(D)** Cell proportion of cell types in GSE163577. **(E)** Circle plot of cell-cell communication network, highlighting astrocyte interactions. **(F)** Heatmap of metabolic pathway activity scores across major cell types.

Analysis of scRNA-seq data from AD hippocampus (GSE163577) after quality control (QC) yielded 33,102 high-quality cells, which clustered into 33 distinct clusters (Figure 5A). Annotation via SingleR identified 7 major cell types, and astrocytes constituted the largest cellular population (Figures 5A–D). CellChat analysis revealed extensive and specific communication networks, with astrocytes acting as a central signaling hub, showing particularly strong interactions with neurons (Figure 5E). Metabolic profiling demonstrated significant heterogeneity, with astrocytes exhibiting a distinct metabolic state in pyruvate and propanoate metabolism (Figure 5F).

### 3.6 Autolysosome signature in astrocyte of AD patients

**FIGURE 6:**
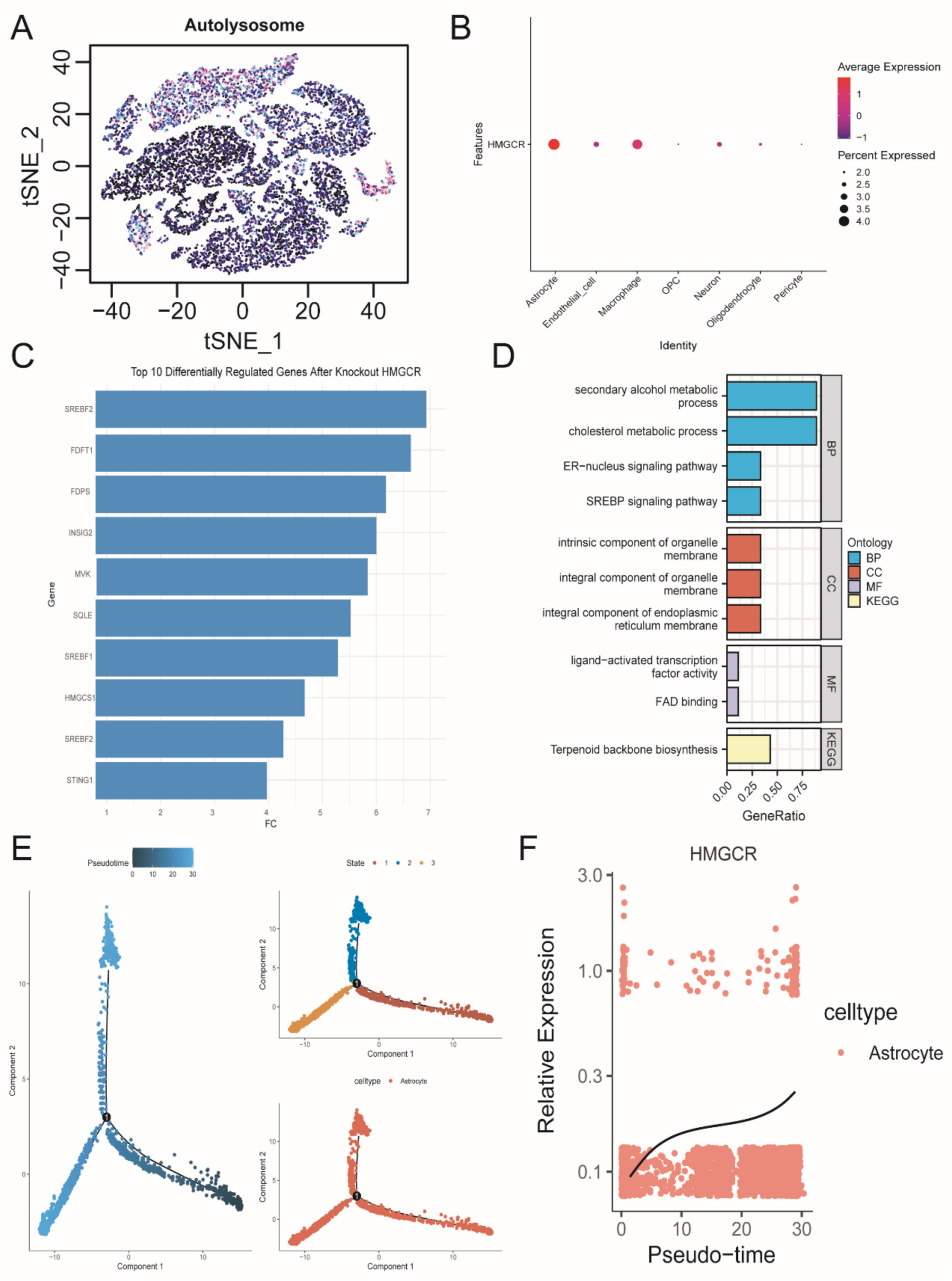
Autolysosome activity and HMGCR function in astrocytes of AD patients. **(A)** AUCELL score of autolysosomes. **(B)** Feature plot showing HMGCR expression on the UMAP. **(C)** Heatmap plot of genes perturbed after virtual HMGCR knockout in astrocytes. **(D)** GO and KEGG enrichment analysis of perturbed genes. **(E)** Pseudotime trajectory analysis of astrocytes. **(F)** HMGCR temporal expression in astrocyte differentiation.

AUCell scoring demonstrated that autolysosome pathway activity was significantly elevated in the astrocyte cluster compared to other cell types (Figure 6A). Feature plot analysis confirmed that HMGCR expression was predominantly localized to astrocytes (Figure 6B). Pseudotime trajectory analysis of astrocytes revealed three distinct differentiation branches of astrocytes, with HMGCR expression remaining persistently high across all trajectories, suggesting its stable role in astrocyte states (Figures 6E,F). An *in silico* virtual knockout of HMGCR specifically within the astrocyte cluster led to significant perturbations in the gene regulatory network. Functional enrichment of the most perturbed genes pointed to pathways involved in functions related to synaptic signaling and cell-cell adhesion (Figures 6C,D).

### 3.7 Drug screening and clinical validation

**FIGURE 7:**
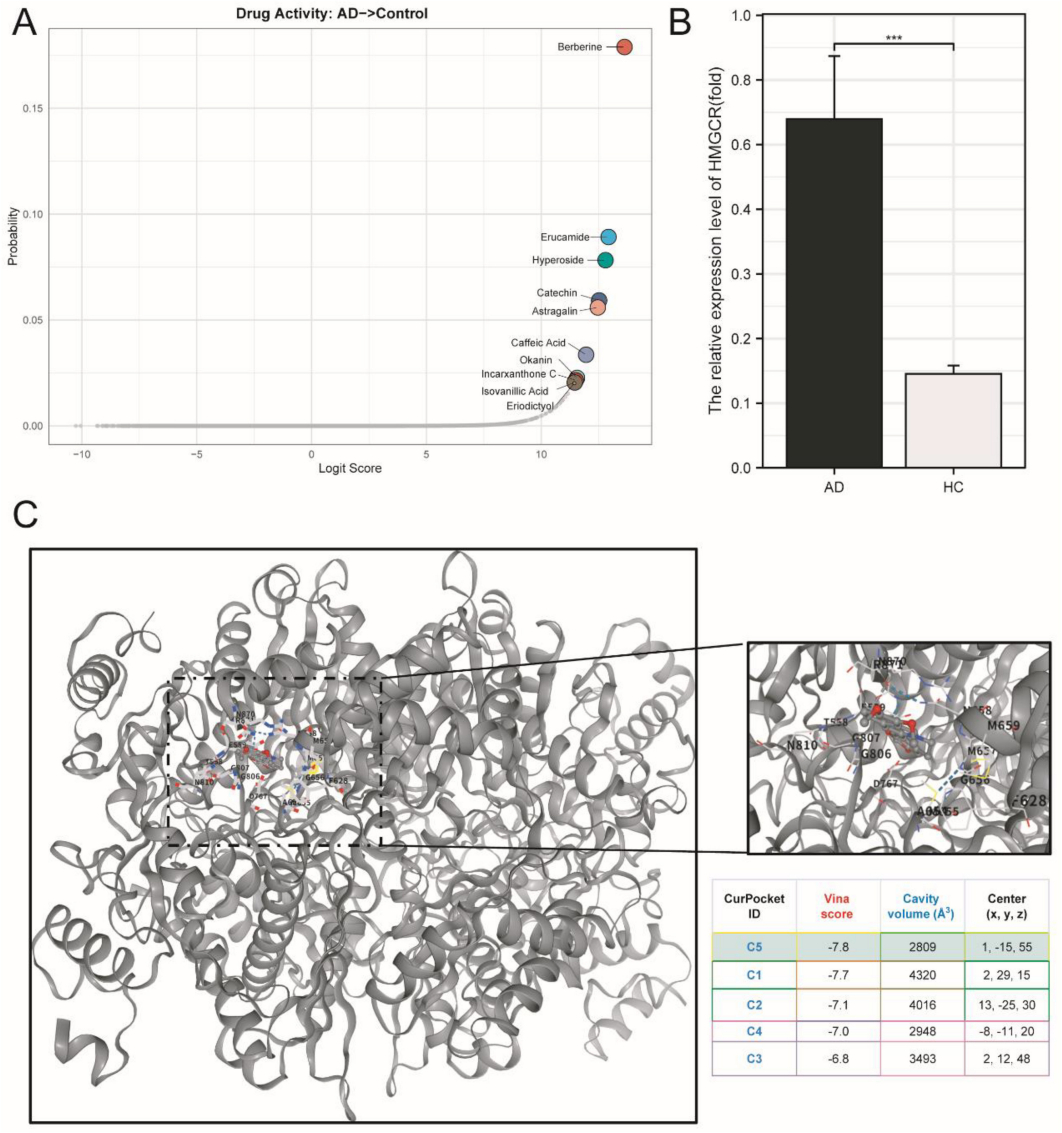
Therapeutic screening and clinical validation in AD patients. **(A)** Workflow and result of the DrugReflector screening identifying Berberine. **(B)** q-RT-PCR quantification of HMGCR protein levels normalized to GAPDH. **(C)** Molecular docking validation.

DrugReflector screening, integrating queries from GSE28146 and the TCMSP database, nominated Berberine as the top-ranking compound with a predicted therapeutic effect on AD (Figure 7A). Molecular docking predicted a stable binding conformation of Berberine within the catalytic site of the HMGCR protein, with a calculated binding energy of -7.8 kcal/mol (Figure 7C). Finally, q-RT-PCR analysis of clinical peripheral samples provided direct translational evidence, showing that HMGCR protein levels were significantly elevated in AD tissues compared to healthy control controls (Figure 7B).

## 4 Discussion and conclusion

This integrative study unveils a novel AA-associated molecular patterns in predictive and therapeutic potentials of AD, with HMGCR emerging as its central regulatory hub pathogenic factor. By harmonizing bulk transcriptomics, single-cell resolution mapping, and clinical data, we have systematically delineated this clinical actionable target.

HMGCR is the rate-limiting enzyme of the mevalonate pathway, primarily governing cellular cholesterol biosynthesis (Yang et al., 2025). Its connection to AD has long been inferred from epidemiological studies suggesting that statins (HMGCR inhibitors) may reduce dementia risk, yet interventional trials have yielded inconsistent results, indicating a nuanced role beyond systemic cholesterol lowering (Zhou et al., 2023). Critically, within the brain, cholesterol homeostasis is largely autonomous, with astrocytes serving as the principal producers (Chang et al., 2017). In AD, reactive astrocytosis is associated with profound alterations in cholesterol metabolism, where an upregulation of HMGCR may drive not only increased cholesterol synthesis but also the production of non-sterol isoprenoids essential for protein prenylation (Zhang W. et al., 2023). This astrocyte-specific metabolic shift can influence key processes, such as dysregulated cholesterol efflux to neurons can impair synaptic membrane remodeling and repair, while aberrant prenylation of small GTPases can destabilize cytoskeletal dynamics and exacerbate pro-inflammatory signaling (Lee et al., 2022). Furthermore, the integrity of the autophagic-lysosomal system is exquisitely sensitive to cholesterol balance (Skeyni et al., 2024). Excessive intracellular cholesterol can accumulate within lysosomal membranes, impairing their hydrolase activity and fusion capacity with autophagosomes, thereby crippling autophagic flux and leading to the accumulation of dysfunctional autolysosomes and pathogenic protein aggregate (Shibuya et al., 2015).

In summary, the core innovation of this work lies in the identification and multi-level validation of AA-associated axis in AD and HMGCR as a lynchpin within a previously unrecognized autolysosome-astrocyte co-expression axis, illuminating a potential pathway through which metabolic dysregulation in astrocytes exacerbates neurodegenerative processes in AD. This study exemplified a potential clinical actionable target in AD. However, the interpretative scope of this study is bound by several limitations. The foundational transcriptomic insights are derived from publicly available datasets, which, despite batch correction, may not fully account for all sources of technical and biological variability. Future studies should combine the clinical information of AD patients, focusing on the estimation of efficacy of diagnostic and molecular stratification models acquired from our study in a large, multi-center clinical trials to enhance the clinical significance. Besides, The single-cell analysis, while offering high-resolution insights, is based on a cohort of 9 AD patients, potentially limiting the capture of the full heterogeneity of astrocyte states across the AD spectrum. Future studies should combined advanced AI technologies in higher resolution single-cell and spatial data of AD patients, coupled with pre-clinical studies, dynamically tracing autolysosome in astrocyte for the pathogenesis of AD patients. In addition, the precise molecular cascades downstream of HMGCR in astrocytes that ultimately impair neuronal health via regulating autolysosome remain to be fully delineated in pre-clinical AD models. Furthermore, Future research should prioritize *in vivo* studies using conditional knockout or astrocyte-specific overexpression of HMGCR in AD mouse models to establish causality and elucidate detailed mechanisms acquired by our *in silico* knockout. Concurrently, high-throughput screening for astrocyte-selective HMGCR modulators could yield more targeted therapeutic leads than existing pan-inhibitors like statins. Similarly, the therapeutic candidate Berberine, identified computationally, demands rigorous experimental testing in preclinical models to confirm its efficacy, optimal dosing, and ability to penetrate the blood-brain barrier and modulate the proposed AA-associated axis and HMGCR *in vivo*.

## Data Availability

The datasets presented in this study can be found in online repositories. The names of the repository/repositories and accession number(s) can be found in the article/supplementary material.
